# Cerebral microdialysis and glucopenia in traumatic brain injury: A review

**DOI:** 10.3389/fneur.2023.1017290

**Published:** 2023-01-27

**Authors:** Himanshu Sharma, John P. McGinnis, Katherine E. Kabotyanski, Shankar P. Gopinath, Jerry C. Goodman, Claudia Robertson, Jovany Cruz Navarro

**Affiliations:** ^1^Department of Neurosurgery, Baylor College of Medicine, Houston, TX, United States; ^2^Baylor College of Medicine, Houston, TX, United States; ^3^Department of Pathology, Baylor College of Medicine, Houston, TX, United States; ^4^Department of Anesthesiology, Baylor College of Medicine, Houston, TX, United States

**Keywords:** cerebral microdialysis, glucopenia, traumatic brain injury, lactate-to-pyruvate ratio, glucose

## Abstract

Traditionally, intracranial pressure (ICP) and partial brain tissue oxygenation (PbtO_2_) have been the primary invasive intracranial measurements used to guide management in patients with severe traumatic brain injury (TBI). After injury however, the brain develops an increased metabolic demand which may require an increment in the oxidative metabolism of glucose. Simultaneously, metabolic, and electrical dysfunction can lead to an inability to meet these demands, even in the absence of ischemia or increased intracranial pressure. Cerebral microdialysis provides the ability to accurately measure local concentrations of various solutes including lactate, pyruvate, glycerol and glucose. Experimental and clinical data demonstrate that such measurements of cellular metabolism can yield critical missing information about a patient's physiologic state and help limit secondary damage. Glucose management in traumatic brain injury is still an unresolved question. As cerebral glucose metabolism may be uncoupled from systemic glucose levels due to the metabolic dysfunction, measurement of cerebral extracellular glucose concentrations could provide more predictive information and prove to be a better biomarker to avoid secondary injury of at-risk brain tissue. Based on data obtained from cerebral microdialysis, specific interventions such as ICP-directed therapy, blood glucose increment, seizure control, and/or brain oxygen optimization can be instituted to minimize or prevent secondary insults. Thus, microdialysis measurements of parenchymal metabolic function provides clinically valuable information that cannot be obtained by other monitoring adjuncts in the standard ICU setting.

## Introduction

Cerebral microdialysis (CMD) is a popular tool for evaluating and managing traumatic brain injury (TBI) in the intensive care unit (ICU). Traditionally, the primary invasive measurements guiding TBI management in patients have been intracranial pressure (ICP) and brain tissue oxygenation. After injury, the brain's metabolic demand increases, which requires more oxidative metabolism of glucose. Simultaneously, however, metabolic and electrical dysfunction can lead to an inability to meet these demands, even in the absence of ischemia or increased ICP. CMD provides the ability to interrogate this derangement by measuring solutes including lactate, pyruvate, and glucose.

CMD involves placing an invasive intraparenchymal monitor into viable brain parenchyma to continuously sample the extracellular fluid milieu. It has been used in clinical settings to evaluate neurochemistry in TBI as well as subarachnoid hemorrhage, prognostication after spontaneous intracranial hemorrhage (1), hypoxic-ischemic encephalopathy (2), and to monitor delivery of chemotherapy (3).

These and other such studies demonstrate that cellular metabolism measurements yield important information about a patient's physiologic state and can help develop new treatment strategies. Targeting metabolic dysregulation could help significantly limit secondary damage because metabolic dysfunction in humans is associated with, and in some cases precedes seizures, ischemia, and increased ICP (4) with feedforward cycles leading to worsening excitotoxicity, mitochondrial dysfunction and impaired autoregulation.

Glucose management in traumatic brain injury is still an unresolved question. Indeed, as of this writing, the Brain Trauma Foundation does not make recommendations regarding serum glucose management in the setting of TBI due to insufficient and contradictory data (5). Metabolic dysfunction may result from derangements in cerebral extracellular glucose concentration, and this may be a biomarker of brain tissue at-risk for secondary injury and/or poor outcome post TBI. Here we review the role of cerebral microdialysis in traumatic brain injury, focusing on the role of cerebral glucose monitoring.

## Cerebral microdialysis

Cerebral microdialysis is a technique that was initially developed as a basic science research tool in the 1960's (6) to more accurately assay alterations in neurochemistry in normal physiology and disease, and later to evaluate the ability of peripherally delivered medications to cross the blood-brain barrier [the early history and development are reviewed by Benveniste (7)].

**Figure 1:**
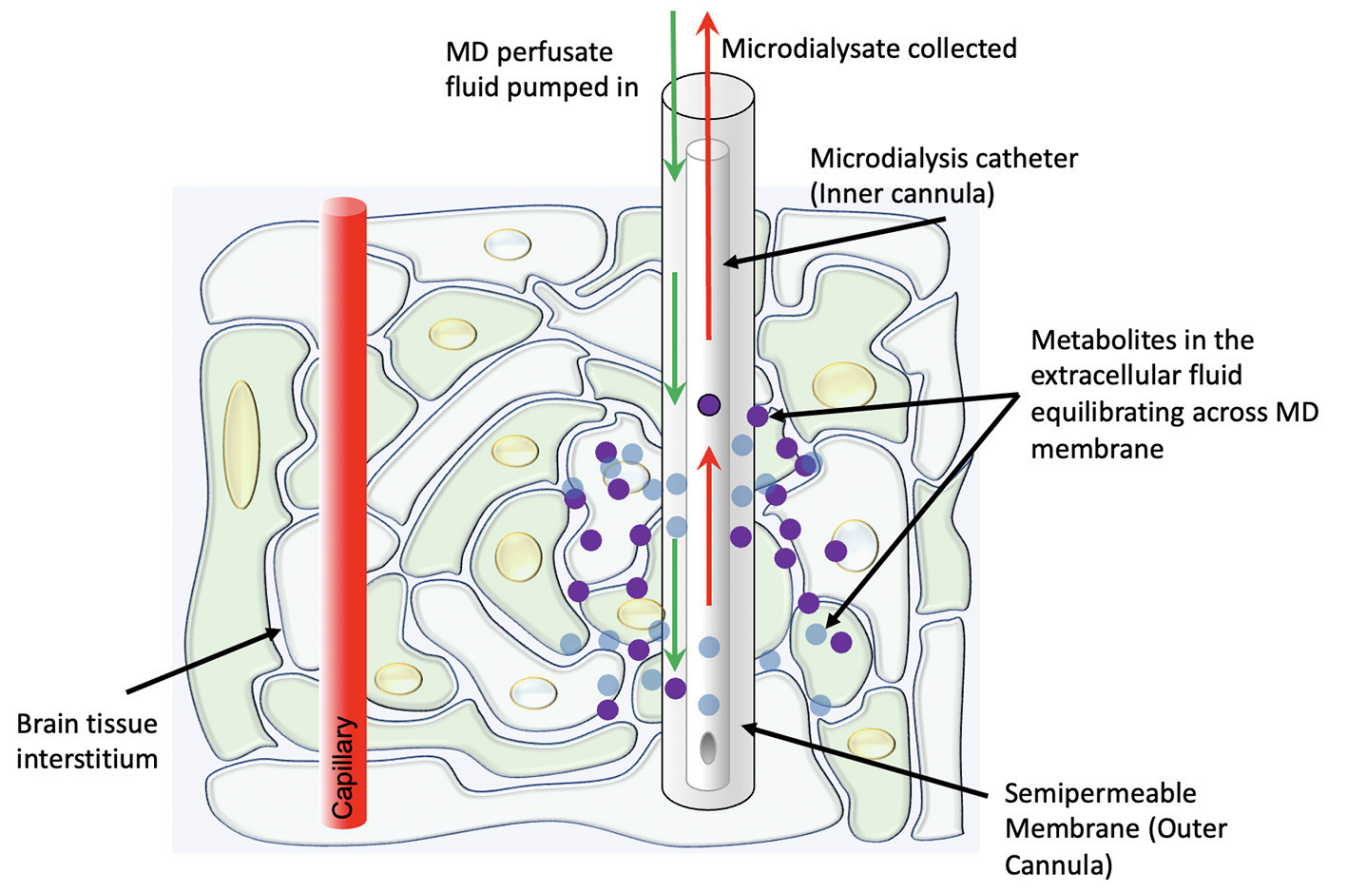
Schematic representation of the physiologic concept behind cerebral microdialysis.

In its modern clinical form, the technique involves the placement of a thin catheter with two concentric lumens into the brain parenchyma, with the inner cannula slightly shorter than the outer cannula (Figure 1). The outer cannula is capped by a semipermeable dialysis membrane which allows for certain metabolites (in clinical use, usually with an upper bound of 20 kDa molecular weight) in the extracellular fluid to enter the catheter. A perfusate fluid is then pumped through the outer cannula and taken up *via* the inner cannula, and metabolites thus sampled from the interstitial fluid are analyzed (8). A plethora of substrates, metabolites, and other chemicals may be measured *via* this method, though glucose, lactate, and pyruvate are currently considered the most clinically informative—“Tier 1”—measurements in the clinical neurocritical care setting (9). Particularly, as an adjunct to intracranial pressure and oxygen tension monitoring, the information from these measurements helps distinguish between ischemic and non-ischemic (e.g., mitochondrial dysfunction) pathophysiologies.

The microdialysis catheter may be placed anywhere in the brain. Often, the catheter is placed in the contralateral hemisphere which can provide information on global metabolism adequacy, and in conjunction with the ICP monitor, can help guide decision making on medical management of ICP to improve oxygen and glucose delivery. However, in the setting of traumatic brain injury, studies have demonstrated that probes placement in “at risk” peri-lesional tissue (i.e., cortical tissue around a focal area of contusion or hematoma) is necessary to detect metabolic changes prior to the wave of global secondary injury (10). It is relevant that brain tissue which appears radiographically normal can nevertheless be significantly injured, remaining at risk, and that placement of a probe into brain tissue that is not salvageable is not useful. Thus, placement of a microdialysis catheter needs to be carefully considered, because while it can provide sustained readings over time, it does so only in a spatially confined region. Additional limitations of microdialysis include the sampling frequency (usually measured hourly) and the low sample volume—both in terms of dialysate (most commonly 0.3 uL/min) as well as spatial restriction.

Recent metabolic imaging studies evaluating blood flow, oxygen/glucose delivery, and oxygen and glucose utilization (11, 12) have demonstrated a great deal of spatial and temporal heterogeneity in metabolic derangements following traumatic brain injury; this work has shed light on ischemic changes that may be present even in the absence of intracranial hypertension and are only inconsistently detected by invasive brain oximetry. Thus, when access to advanced metabolic imaging [e.g., 15O and 18FDG PET, proton, and phosphorus spectroscopy (13)] is available, invasive and non-invasive monitoring can help reveal the extent of metabolic derangement seen after TBI, which can help guide clinical decision making as well as highlight potentially fruitful avenues for the development of therapeutics.

## Relevance of invasive neurochemical monitoring

Cerebral microdialysis can provide several critical pieces of clinically relevant information that are not obtainable by other means in the standard ICU setting in TBI patients. These include a direct measurement of parenchymal metabolic state, under certain circumstances, global cerebral catastrophe (i.e., ICP elevation and metabolic failure) prior to onset, information about seizures and epileptiform discharges not detectable by surface electroencephalogram (EEG), and direct detection of metabolic dysfunction in the absence of ischemia. Combined with other forms of invasive monitors such as ICP and brain tissue oxygen monitoring, this information can help guide clinical decision-making when viable treatment options exist.

Glucose, lactate, and pyruvate as well as the secondarily calculated lactate:pyruvate ratio (LPR) convey information about the availability of substrate for adenosine triphosphate (ATP) generation as well as the redox state of the local tissue. Lactate conversion to pyruvate (an oxidative process) can be impaired by either mitochondrial injury or lack of oxygen availability and thus the LPR is a readout of metabolic stress.

Interestingly, microdialysis of peri-contusional brain in patients with fatal traumatic brain injury (as opposed to “normal” brain distant from site of injury) demonstrated changes in cerebral energy metabolism *prior to* subsequent increase in intracranial pressure (10). Furthermore, in 43% of patients, epileptiform discharges were detectable only by depth electrode monitoring and not the clinically more frequently used surface EEG. Nonetheless, these episodes were associated with metabolic crisis [as defined by elevated LPR, and which have been associated with worsened outcomes (14–16)], detectable by microdialysis catheters, and patients who demonstrated seizures and epileptiform discharges could be distinguished from those who did not by their cerebral metabolic profiles (17). The relationship between epileptic activity and cerebral metabolic changes in the context of TBI is difficult to elucidate as metabolic changes can lead to epileptic activity, but the reverse is also certainly true. While the causal links between metabolic crises and epileptic activity remain an active area of investigation, it is possible that cerebral microdialysis may function as a less invasive measure than invasive EEG to identify patients who may benefit from additional antiepileptic treatment thereby potentially reducing secondary damage by improving ICP and the cerebral metabolic profile.

Additionally, experimental models in pigs have confirmed that various states of cerebral metabolic dysfunction—specifically with altered redox balance—can occur in the absence of cerebral ischemia. This dysfunction is detectable through elevations in lactate and the lactate/pyruvate ratio as measured by microdialysis (18). These studies have been validated in humans with TBI wherein the vast majority of these cerebral metabolic crises occur in the absence of a concomitant reduction in PbO_2_ readings (19). Additional clinical TBI studies using PET in conjunction with tissue PbO_2_ monitoring have noted evidence of tissue hypoxia in the absence of classical ischemia as defined by a reduction in cerebral blood flow and increase in oxygen extraction fraction (OEF). These findings are suggestive of an increased diffusion barrier preventing cellular oxygen delivery and are consistent with microvascular ischemia. Such findings are a further mechanism responsible for metabolic dysfunction consistent with metabolic crisis (20, 21).

Indeed, beyond its use in traumatic brain injury, cerebral microdialysis can give valuable information about neurologic compromise in other patients in the ICU setting. Elevated lactate levels and LPR can predict delayed cerebral ischemia in subarachnoid hemorrhage (22). Microdialysis has also been used to inspire and validate the use of new therapeutics and guide clinical treatment strategies in neurologic ICU patients (23–25).

Thus, focal microdialysis measurements of parenchymal metabolic function provide clinically valuable information continuously at the bedside that cannot be obtained by other monitoring adjuncts in the standard ICU setting.

## Parenchymal glucose in traumatic brain injury

**Figure 2:**
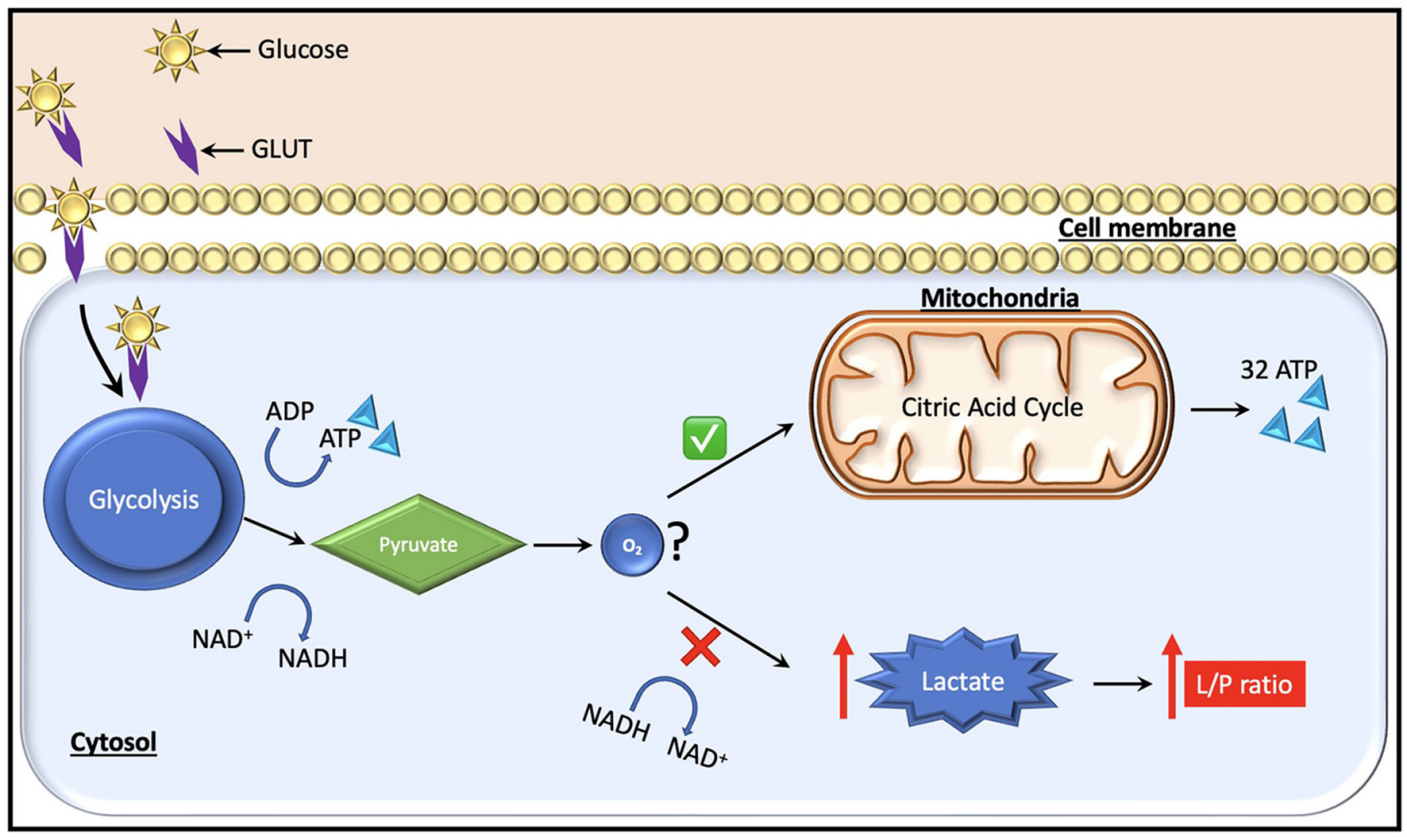
Glucose, which is the major fuel in the brain, is transported across the cell membranes by facilitated diffusion mediated by glucose transporter proteins (GLUT). Two types of glucose transporters (sodium dependent and facilitative transporters) are localized in the membranes of neurons, astrocytes and brain endothelial cells. Of these, GLUT-1 and GLUT-3 represent the two most important transporters in the brain. Under normal conditions, pyruvate is formed from glucose after entering the glycolytic process generating two molecules of ATP. Subsequently, it enters the citric acid cycle if oxygen is available, yielding 32 ATP molecules. During a state of cerebral hypoxia and low glucose (e.g., intracranial hypertension) the production of ATP from the citric acid cycle decreases. Then, cells attempt to compensate for the decrease in ATP production by increasing the turnover of glucose in the anaerobic part of the glycolysis. During this process it is necessary to regenerate NAD+ from NADH, which facilitates maintenance of glycolysis. The NAD+ regeneration causes an increase in lactate and the lactate/pyruvate ratio. The ratio is essentially the same in all tissues (i.e., about 20). A ratio above 25 is considered as an early warning of beginning metabolic crises.

In the context of normal aerobic physiology, glucose is the predominant substrate (~95% of ATP generation) used to meet the metabolic requirements of the brain. An alternate energy source for neurons, particularly after injury, is lactate, which is produced by astrocytes and taken up by neurons as needed as per the astrocyte-neuron lactate shuttle hypothesis (26). Recent studies using CMD have also highlighted the use of ketone bodies as an energy source in the injured brain (27). Under anaerobic conditions on the other hand, glucose can no longer be oxidized to generate ATP, and instead glycolysis predominates with the generation of ATP, lactate, hydrogen ions, and pyruvate as glycolytic end products (Figure 2). Cerebral glucose metabolism in TBI is complex and, while beyond the scope of this review, have been reviewed elsewhere (13, 28). Certain key points bear discussion. Initially after TBI, cerebral glucose intake transiently increases without a rise in oxygen uptake—this has been suggested to be related to hyperglycolysis and/or the generation of reducing equivalents *via* the pentose phosphate pathway shunt which may provide alternate energy sources and reduce oxidative stress in the immediate post-injury period [reviewed by Zhou and Kalanuria (8)]. Subsequently, a global depression in glucose metabolism often predominates.

The etiology of this global decrease in extracellular glucose availability remains a matter of debate, and may include a switch to lactate as a more preferred energy substrate in the injured brain [reviewed by Carpenter et al. (29)], but is regardless associated with poorer long-term outcomes (30). As mentioned previously, the role of clinical management of cerebral glucose levels remains unclear, since studies have demonstrated seemingly conflicting data. Table 1 highlights key papers studying the relationship between glucose control and clinical outcomes in TBI.

**Table 1:** Key papers studying the role of glucose levels and/or glycemic control parameters after traumatic brain injury and clinical and physiological outcome measures.

**References**	**Title**	**Type**	**Aims**	**Conclusions**
Timofeev et al. (14)	Cerebral extracellular chemistry and outcome following traumatic brain injury: A microdialysis study of 223 patients	Observational study	Determine the relationship between the fundamental biochemical markers and neurological outcome in a large cohort of patients with traumatic brain injury	Significant association of higher lactate/pyruvate ratio with increased mortality and unfavorable outcomes after TBI
Hattori et al. (30)	Correlation of regional metabolic rates of glucose with glasgow coma scale after traumatic brain injury	Observational study	Compare the regional cerebral metabolic rate of glucose with the severity of injury and level of consciousness in adults with TBI and healthy controls	Significant difference in glucose metabolism between comatose and non-comatose patients acutely after TBI; the metabolic rate of glucose in these regions significantly correlated with the level of consciousness at the time of PET
Oddo et al. (31)	Impact of tight glycemic control on cerebral glucose metabolism after severe brain injury: A microdialysis study	Observational study	Analyze the effect of tight glycemic control with the use of intensive insulin therapy on cerebral glucose metabolism in patients with severe brain injury	In patients with severe brain injury, tight systemic glucose control is associated with reduced cerebral extra-cellular glucose availability and increased prevalence of brain energy crisis, which in turn correlates with increased mortality
Goodman et al. (32)	Extracellular lactate and glucose alterations in the brain after head injury measured by microdialysis	Observational study	Study cerebral glucose and lactate metabolism in 126 head-injured patients using microdialysis	Increased lactate and decreased glucose, indicating accelerated glycolysis, commonly occurred with cerebral ischemia or hypoxia is associated with a poor outcome
Vespa et al. (33)	Persistently low extracellular glucose correlates with poor outcome 6 months after human traumatic brain injury despite a lack of increased lactate: A microdialysis study	Observational study	Assess whether posttraumatic reductions in extracellular glucose levels are due to ischemia and are associated with poor outcomes	The level of extracellular glucose is typically reduced after traumatic brain injury and total duration of low glucose is associated with poor outcome, but not with ischemia
Vespa et al. (34)	Intensive insulin therapy reduces microdialysis glucose values without altering glucose utilization or improving the lactate/pyruvate ratio after traumatic brain injury	Observational study	Determine whether intensive glycemic control using insulin results in reduced brain extracellular glucose and glucose metabolic rates after TBI; compare the effects of intensive vs. loose glycemic control on clinical outcome and incidence rate of microdialysis markers of cellular distress	Intensive glycemic control did not reduce global cerebral metabolic rate compared with routine glycemic control; intensive glycemic control resulted in reduced microdialysis glucose concentrations and increased microdialysis markers of brain metabolic distress (LPR and glutamate); no differences found in mortality rate or functional outcome between intensive and loose glycemic control
Meier et al. (35)	Differential temporal profile of lowered blood glucose levels (3.5–6.5 vs. 5–8 mmol/l) in patients with severe traumatic brain injury	Observational study	Assess whether maintaining arterial blood glucose between 3.5 and 6.5 mmol/l, as compared with 5 to 8 mmol/l, significantly decreases mortality and reduces rates of infectious complications in patients with severe TBI	High serum glucose levels in the 1st week after injury and reduced glucose targets thereafter are associated with improved outcomes, including reduced instances of elevated ICP
Rostami (36)	Glucose and the injured brain-monitored in the neurointensive care unit	Review	Review studies reporting on monitoring of cerebral glucose with microdialysis in patients with traumatic brain injury (TBI), subarachnoid hemorrhage (SAH) and ischemic stroke	Low cerebral glucose in patients with TBI and SAH provides valuable information on development of secondary ischemia and has been correlated with worse outcome
Rajagopalan et al. (37)	Hierarchical cluster analysis identifies distinct physiological states after acute brain injury	Observational study	Test whether data-driven approaches can identify distinct physiological states from intracranial multimodality monitoring data	Patients with a favorable outcome had a greater proportion of physiologically normal events, whereas patients with an unfavorable outcome had a greater proportion of ischemia and hyperglycolysis
Jalloh et al. (38)	Focally perfused succinate potentiates brain metabolism in head injury patient	Observational study	Investigate whether focal administration of succinate, a tricarboxylic acid cycle intermediate, could improve cerebral metabolism in TBI patients	Infusion of succinate *via* cerebral microdialysis catheter improved glucose utilization and decreased LPR in acute TBI
Stovell et al. (39)	The effect of succinate on brain NADH/NAD+ redox state and high energy phosphate metabolism in acute traumatic brain injury	Observational study	Study the effect of microdialysis-delivered succinate on brain energy state (phosphocreatine/ATP ratio) and tissue NADH/NAD+ redox state (L/P ratio) using microdialysis in patients with acute major TBI	Succinate improves NADH/NAD+ redox state (decreases L/P ratio) in the traumatized human brain; a significant correlation between percentage decrease in L/P ratio and percentage increase in PCr/ATP suggests that succinate supplementation can increase brain energy metabolism

In traumatic brain injury, increased cerebral glucose is associated with increased serum glucose (40) and increased mortality (14) which is consistent with the currently accepted understanding that hyperglycemia in surgical patients is associated with poorer outcomes. However, a key caveat in neurologically injured patients is that when specifically evaluating injured and non-injured hemispheres in TBI patients, only the non-injured hemisphere demonstrated a correlation between serum and cerebral glucose levels, while the injured hemisphere had no correlation between the two (41). Furthermore, reduction of plasma glucose with insulin does, as expected, decrease cerebral glucose. However, an intensive approach to glucose control is associated with brain energy crises in TBI patients and has conflictingly been described to be (31–33), and not specifically to be (34), associated with worsened outcomes in TBI patients.

Thus, it is likely that a more nuanced approach is necessary in managing cerebral glucose levels as the details of these studies indicate a heterogeneity in the causes and effects of cerebral glucopenia. Indeed, in one study glucopenia predicted poor outcomes even in the absence of increased lactate or lactate/pyruvate ratio, suggesting that the poorer outcomes predicted by cerebral glucopenia are not due to cerebral ischemia (33). Clinical TBI studies combining 15O and 18FDG PET in conjunction with microdialysis have noted that low extracellular glucose was related to dysfunctional glucose delivery, primarily due to reduced cerebral blood flow, leading to increases in the rate of glycolysis. This may act as a potent mechanism of secondary injury (12, 13). Additionally, while intensive insulin therapy reduced cerebral glucose in TBI patients, it worsened glutamate levels and the lactate/pyruvate ratio, and ultimately did not alter mortality or 6-month clinical outcome (34). Thus, cerebral glucose levels likely exert an impact on brain metabolism and function in both redox state dependent and independent manners. Additionally, a study evaluating various serum glucose targets (without cerebral microdialysis) suggested that high serum glucose levels in the 1st week after injury and reduced glucose targets thereafter are associated with improved outcomes including reduced instances of elevated ICP, suggesting that a temporal component may also be critical to understanding serum and cerebral glucose roles and optimal management strategies after traumatic brain injury (35).

Overall, as reviewed by Rostami (36), while hyperglycemia is associated with poorer outcomes in TBI patients, tight glycemic control does not consistently demonstrate a significant improvement in those outcomes and precipitates cerebral metabolic dysfunction, with some evidence that subsequent glucopenia and metabolic crisis may be associated with worse outcomes.

However, emerging data indicate that there are various causes of alterations in cerebral glucose metabolism after TBI, and a deeper understanding of these underlying factors needs to be sought. Variations in neuronal uptake of astrocyte-produced lactate as a preferred energy source as well as the presence of seizure activity need to be considered when managing TBI patients in the ICU. Clearly, however, without cerebral microdialysis information, the remaining balance of intra- and extracranial measurements of metabolism commonly used is insufficient to fully understand parenchymal tissue function and omits key predictive information from the physician's repertoire of data.

## Current and ongoing directions

More recently, multimodal hierarchical cluster analyses utilizing cerebral microdialysis have begun to delineate neurologic “events” differentiating for example, between hyperglycolysis, ischemia, and pure ICP elevations each correlated with different outcome profiles (37).

Excitingly, the use of cerebral microdialysis has permitted direct interrogation and manipulation of parenchymal tissue metabolism. This opens the possibility of targeting, either by systemic or more focused interventions, certain levels of metabolism in a manner similar to our targeting of brain oxygenation. Further work will need to clarify whether such targeting can improve clinical outcomes. In one recent study, infusion of succinate (a TCA cycle intermediate) *via* the CMD catheter improved glucose utilization and decreased LPR as well as extracellular glutamate (38, 39).

There is burgeoning interest in the use of CMD to guide nutrition in TBI patients, though this field is still in its infancy (4, 42). Evidence of ketone body utilization and the possibility of ketogenic diet for TBI patients (27) and the development of hypertonic lactate as a therapeutic in TBI (23) are some ongoing avenues of research in this field, and likely with more data, more optimized nutritional strategies may be formulated for TBI patients.

Perhaps less intuitively, systemic complications may also be reflected by intracranial metabolism after traumatic brain injury. For example, early elevation of cerebral lactate levels correlated positively with the development of pneumonia in patients with aneurysmal subarachnoid hemorrhage, whereas delayed elevation in lactate correlated with delayed ischemic neurologic deficits, highlighting potential links with immune regulation (43).

Thus, while the field remains in its infancy at the moment, direct measurement of intraparenchymal metabolic function *via* cerebral microdialysis has shed light on a wide variety of hitherto unexplored mechanisms underlying dysfunction and recovery in traumatic brain injury. Glucose metabolism in particular is dysregulated in this state, and metabolic stress in patients with glucopenia is likely mediated through both ischemic and non-ischemic mechanisms. The relationship between cerebral glucose levels and recovery, systemic physiologic events, and long-term neurologic outcomes is complex, and merits further investigation.

## Author contributions

All authors contributed to manuscript revision, read, and approved the submitted version.
